# Heat shock protein 70 promotes lipogenesis in HepG2 cells

**DOI:** 10.1186/s12944-018-0722-8

**Published:** 2018-04-10

**Authors:** Jielei Zhang, Nengguang Fan, Yongde Peng

**Affiliations:** Department of Endocrinology and Metabolism, Shanghai General Hospital, Shanghai Jiao Tong University School of Medicine, 100 Haining, Road, Shanghai, 200080 China

**Keywords:** Heat shock protein 70, Fatty acid synthase, Non-alcoholic fatty liver disease, Stearoyl-CoA desaturase, Acetyl-CoA carboxylase

## Abstract

**Background:**

The increasing prevalence of non-alcoholic fatty liver disease (NAFLD) has followed the international rise in obesity rates. Multiple mechanisms are involved in NAFLD, including endoplasmic reticulum stress and oxidative stress. Heat shock protein 70 (HSP70), which is abundant in most organisms, is sensitive to stress. However, the role of HSP70 in NAFLD has not been investigated. Here, we investigated the possible role of HSP70 in lipid synthesis.

**Methods:**

C57BL/6 mice were fed a high-fat diet, and HepG2 cells were treated with 0.5 mM palmitic acid (PA). HSP70 expression was detected by qPCR, Western blot and immunohistochemistry. Total cholesterol (TC) and triglyceride (TG) levels were detected by enzyme-linked immunosorbent assay (ELISA). After Hsp70 overexpression and knockdown, TC and TG levels and FAS, SCD, and ACC expression were detected.

**Results:**

HSP70 expression was significantly increased in the livers of obese mice. In vitro, HSP70 expression was markedly induced by PA in HepG2 cells. Notably, HSP70 overexpression in HepG2 cells enhanced TC and TG synthesis, in parallel with the upregulation of lipogenic genes, including FAS, SCD and ACC. By contrast, HSP70 knockdown decreased the levels of cellular lipids and the expression of FAS, SCD, and ACC in HepG2 cells. Together, our results suggest that HSP70 may promote lipogenesis in HepG2 cells.

**Conclusions:**

Heat shock protein 70 promotes lipogenesis in HepG2 cells.

## Background

NAFLD is one of the most common causes of chronic liver disease and is distinguished by the superabundant deposition of lipids in hepatocytes of the liver parenchyma [1]. Multiple mechanisms are involved in the development of NAFLD, including endoplasmic reticulum (ER) stress and oxidative stress. Heat shock proteins (HSPs) are triggered by heat stress and other short-term and long-term stresses. Heat shock protein 70 is the most well-studied in response to stress and has been identified as a significant modulator of adaptation during thermal stress in domestic animals [2]. The role of HSP70 in stress reactions varies, and research findings have been inconsistent. This may be because HSP70 has dual roles; iHSP70 is anti-inflammatory, while eHSP70 plays a pro-inflammatory role and is correlated with insulin resistance and beta cell dysfunction in patients with diabetes [3]. LPS-induced fever results in simultaneous increases in HSP70 and eHSP70, which could trigger pro-inflammatory eHSP70 expression in human macrophages, but the underlying mechanism is unknown [4]. The heat shock response (HSR) and HSP90 play important roles in the development of NAFLD. Moreover, HSP90 overexpression in liver cell lines increases neutral lipid accumulation [5]. Heat stress induces hepatic lipogenesis in chickens, and this effect is likely mediated by HSPs [6]. However, the specific role of HSP70 in NAFLD remains unclear.

In view of these considerations, we studied the changes in HSP70 expression in mice with NAFLD and the effect of HSP70 on hepatocyte lipogenesis. We demonstrated that HSP70 expression is increased under a high-fat dietary regime using a mouse model of NAFLD. In addition, our in vitro overexpression experiments showed that HSP70 could promote lipogenesis.

## Methods

### Animal experiments

All mice used were male C57BL/6 mice weighing 18–22 g. The mice were fed either standard normal feed (NFD,10% kcal from fat, *n* = 5) (Research Diets, New Brunswick, USA) or a high-fat diet (HFD, 60% kcal from fat, *n* = 5) (Research Diets, New Brunswick, USA) for 16 weeks. The animals were treated according to high ethical and scientific standards with oversight by the Animal Center at Shanghai General Hospital.

### Immunohistochemistry

Paraffin-embedded sections were deparaffinized and rehydrated. The sections were then prepared conventionally by boiling in citrate buffer for 20 min and were subsequently incubated in 3% H_2_O_2_ for 10 min to eliminate endogenous peroxidase activity. The samples were then rinsed with phosphate-buffered saline (PBS), blocked with goat serum for 15 min, and washed again with PBS. An anti-Hsp70 antibody (1:100, Cell Signaling Technology Co. USA) was added to the sections, which were incubated overnight at 4 °C. The next day, the sections were washed with PBS before a biotinylated secondary antibody was added to the slides. After a second wash with PBS, horseradish-peroxidase-labelled streptavidin was added. The slides were rinsed with PBS before DAB and haematoxylin were applied. The samples were dehydrated with ethanol, mounted, and examined by microscopy. For the negative control, the slides were incubated with PBS instead of primary antibody.

### Modelling of NAFLD in HepG2 cells

The HepG2 cells used in this study were obtained from ATCC (USA). The cells were cultured in medium (GIBCO Company, USA) with 10% FBS (Control Group, Australia). The cells were maintained in an incubator with 5% CO_2_ and saturated humidity conditions, and the temperature was set at 37 °C.The cells grew to 80% confluence in 2 days, at which point the culture medium was replaced.

### Palmitic acid-induced steatosis

Steatosis was induced using previously described methods with slight modification. After reaching 75% confluence, Control cells and cells with stable HSPA1A overexpression or knockdown were serum-starved for 12 h and then the high-fat group was exposed to 0.5 mM PA was prepared in culture media containing 1% fatty acid-free bovine serum albumin (BSA),while the cells in normal group treated with 1% BSA. Gene expression was evaluated after 24 h of incubation with PA.

### Oil red O staining

The culture medium was completely removed, and the cells were rinsed with PBS. The cells were fixed with an appropriate solution for 30 min at room temperature and then rinsed twice with PBS gently. Freshly prepared Oil Red O working solution (Abcam, England) was added, and the cells were incubated for 60 min at room temperature. The staining solution was removed, and the cells were washed with PBS 2-3 times. The cells were then visualized under a light microscope, and images were captured.

### Quantitative PCR analysis

TRIzol reagent (Invitrogen, Carlsbad, California, USA) was used to extract total RNA from HepG2 cells. A reverse transcription kit (TaKaRa, Dalian, China) was used to transcribe cDNA from 500 ng of RNA, and a SYBR Green Supermix Kit (Bio-Rad, Hercules, California, USA) was used for quantitative real-time PCR with the cDNA template. The primer sequences used for PCR amplification are shown in Table 1.

**Table 1:** Primer sequences for Quantitative PCR.

HSPA1A	Sense 5′-3’	CCACCATTGAGGAGGTAGATTAG
	Antisense 5′-3’	TCATCTCTGCATGTAGAAACCG
FAS	Sense 5′-3’	AAGGACCTGTCTAGGTTTGATGC
	Antisense 5′-3’	TGGCTTCATAGGTGACTTCCA
SCD	Sense 5′-3’	GCCCCTCTACTTGGAAGACGA
	Antisense 5′-3’	AAGTGATCCCATACAGGGCTC
ACC	Sense 5′-3’	TCACACCTGAAGACCTTAAAGCC
	Antisense 5′-3’	AGCCCACACTGCTTGTACTG
PPARα	Sense 5′-3’	ATGGTGGACACGGAAAGCC
	Antisense 5′-3’	CGATGGATTGCGAAATCTCTTGG
SREBP1C	Sense 5′-3’	CGGAACCATCTTGGCAACAGT
	Antisense 5′-3’	CGCTTCTCAATGGCGTTGT
ACOX	Sense 5′-3’	GGAACTCACCTTCGAGGCTTG
	Antisense 5′-3’	TTCCCCTTAGTGATGAGCTGG
CPT-1	Sense 5′-3’	TCCAGTTGGCTTATCGTGGTG
	Antisense 5′-3’	TCCAGAGTCCGATTGATTTTTGC
GAPDH	Sense 5′-3’	CCATGTTCGTCATGGGTGTGAACCA
	Antisense 5′-3’	GCCAGTAGAGGCAGGGATGATGTTC

### Determination of TG and TC levels in the supernatant of HepG2 cells

The TG and TC levels were determined using an enzyme-linked immunosorbent assay (ELISA) kit (USCN Life Science, Wuhan, China) according to the manufacturer’s instructions. The absorbance of the samples was measured at 450 nm (Bio-Tek ELX800, Winooski, USA).

### Western blot analysis

A mammalian protein extraction reagent was used to extract protein, and the protein concentration was determined using the Bradford method. The samples were subjected to SDS-PAGE and electrotransferred to a polyvinylidene fluoride membrane for 2 h. After transfer, the membrane was incubated with an anti-Hsp70 antibody (1:200, Cell Signaling Technology Co. USA) overnight at 4 °C and then with a secondary antibody for 1 h, followed by film exposure and development. The film was scanned immediately. The Western blot results were evaluated using Image-Pro Plus image analysis software.

### Production of HSPA1A overexpression or knockdown lentiviruses

HEK293T cells were seeded in 10-cm culture dishes. When the cells were 90% confluent, they were transfected using Lipofectamine 2000 (Invitrogen). Briefly, each dish was transfected with 5 μg of a lentivirus vector containing the target cDNA, 5 μg of a helper plasmid containing Gag/Pol/Rev. (Helper 1) and 2.5 μg of a helper plasmid containing the VSVG envelope. Culture supernatants were collected at 24 h and 48 h post transfection. The virus particles were concentrated by ultracentrifugation (Beckman Optima L-90 K, type 50.2 rotor, 50,000 rpm, 3 h). HSPA1A deletion mutants and Plk4 coding sequences were cloned into the pcDNA3.0 vector, pLVX-IRES-PURO-GFP vector, or pLenti-GFP vector by standard cloning methods. Double-stranded shRNA oligonucleotides targeting HSPA1A were cloned into pLKO.1 (with or without GFP), and the sequences are as follows:

HSP70-sh1 5’-CCGGGCCTTTCCAAGATTGCTGTTTCTCGAGAAACAGCAATCTTGGAAAGGCTTTTTG-3′;

HSP70-sh2 5’-CCGGTCAATTTCCTGTGTTTGCAATCTCGAGTCAATTTCCTGTGTTTGCAATTTTTTG-3′;

HSP70-sh3 5’-CCGGCTGTTTGTCAGTTCTCAATTTCTCGAGAAATTGAGAACTGACAAACAGTTTTTG-3’.

### Lipid accumulation and measurement

Cells were incubated in the absence or presence of 0.5 mM PA in the culture medium. The TC and TG levels in the supernatants of HepG2 cells were determined by ELISA (ZCI BIO, Shanghai, China). For lipid droplet staining, the cells were fixed with 10% formalin, stained with Oil Red O working solution and visualized under a bright-field microscope. Alternatively, DAPI (0.1 μg/ml) in PBS was applied to stain lipid droplets for 2 min and visualized by fluorescence microscopy.

### Statistical analysis

Student’s two-tailed t-test and GraphPad Prism were used for statistical analysis. The results in each group were expressed as the mean ± SD, and *p* < 0.05 was considered significant.

## Results

### HSP70 expression is up-regulated in fatty liver

**Fig. 1:**
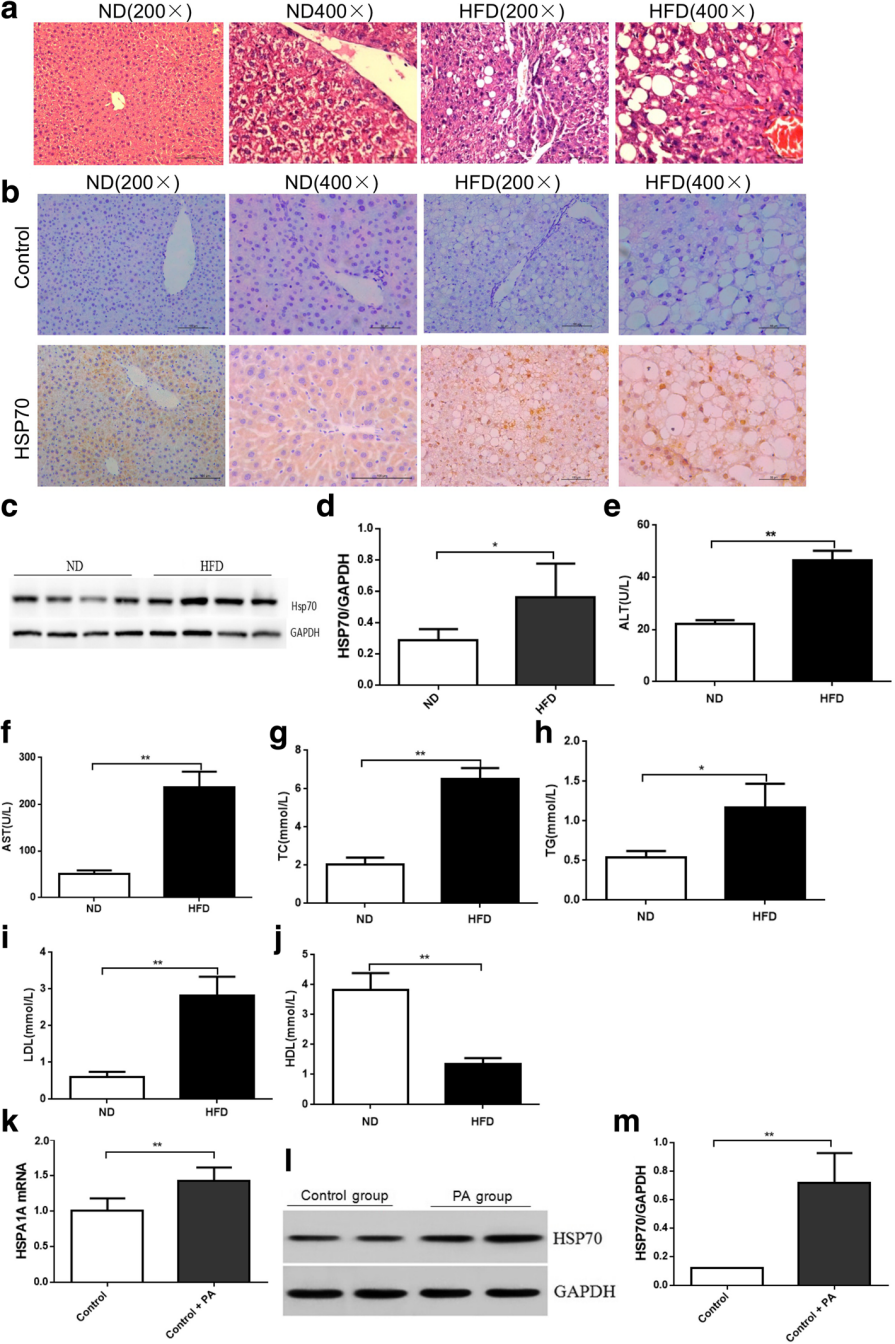
Hsp70 expression in the liver and in HepG2 cells. Histopathological changes in the livers of mice in the ND group and HFD group (**a** HE staining, original magnification × 200; × 400). Hsp70 expression in each group. HSP70 expression increased and localized to the cell nucleus in the HFD group compared with the control group (**b**-**c**). HSP70 protein expression clearly increased in the HFD group compared with the ND group (*P* < 0.05). Effects of high fat on HepG2 cells. Oil Red O staining after 24 h of treatment indicates the presence of few lipid droplets in the control group, whereas there were many lipid droplets in the PA-treated group (**d** original magnification × 200; × 400). In HepG2 cells, HSP70 mRNA and protein expression clearly increased in the PA group compared with the control group (*P* < 0.05). HSP70 mRNA (**e**) and protein (**f**) expression levels were evaluated by qPCR and Western blot and normalized to GAPDH levels. Data are expressed as the mean ± SD (*n* = 6, each). Blood samples were then collected, and the levels of ALT (**e**), AST (**f**), TC (**g**), TG (**h**), LDL (**i**), and HDL (**j**) in the plasma were determined. Data represent the mean from six animals, and error bars indicate the SD. *P < 0.05 compared with the control group, ** *P* < 0.01 compared with the control group

To examine the association between HSP70 and fatty liver, we first evaluated the expression of HSP70 in the livers of obese mice. Six-week-old mice were fed a HFD or normal diet for 16 weeks. As shown in Fig. 1a, mice with high-fat diet-induced obesity displayed liver steatosis (Fig. 1a). As expected, the protein expression of HSP70 in the liver was up-regulated in obese mice compared with control mice (Fig. 1b,c), as assessed by Western blot and immunohistochemistry. ALT, AST, TC, TG, LDL and HDL concentrations in plasma were detected by testing blood samples (Fig. 1e-j). Compared with the ND group, ALT, AST, TC and LDL were significantly increased (*P* < 0.01) and TG also increased (*P* < 0.05) in the HFD group. HDL was significantly lower in the HFD group than in the ND group (*P* < 0.01).

To confirm the in vivo results in vitro, HepG2 cells were treated with 1% BSA or 0.5 mM PA for 24 h. Oil Red O staining was used to observe intracellular lipid droplets in the HepG2 cells (Fig. 1d). As shown in Fig. 1, PA induced lipid accumulation in HepG2 cells. Moreover, HSP70 mRNA and protein were up-regulated in PA-treated HepG2 cells (Fig. 1e-f).

### HSPA1A overexpression promotes lipid accumulation in HepG2 cells

**Fig. 2:**
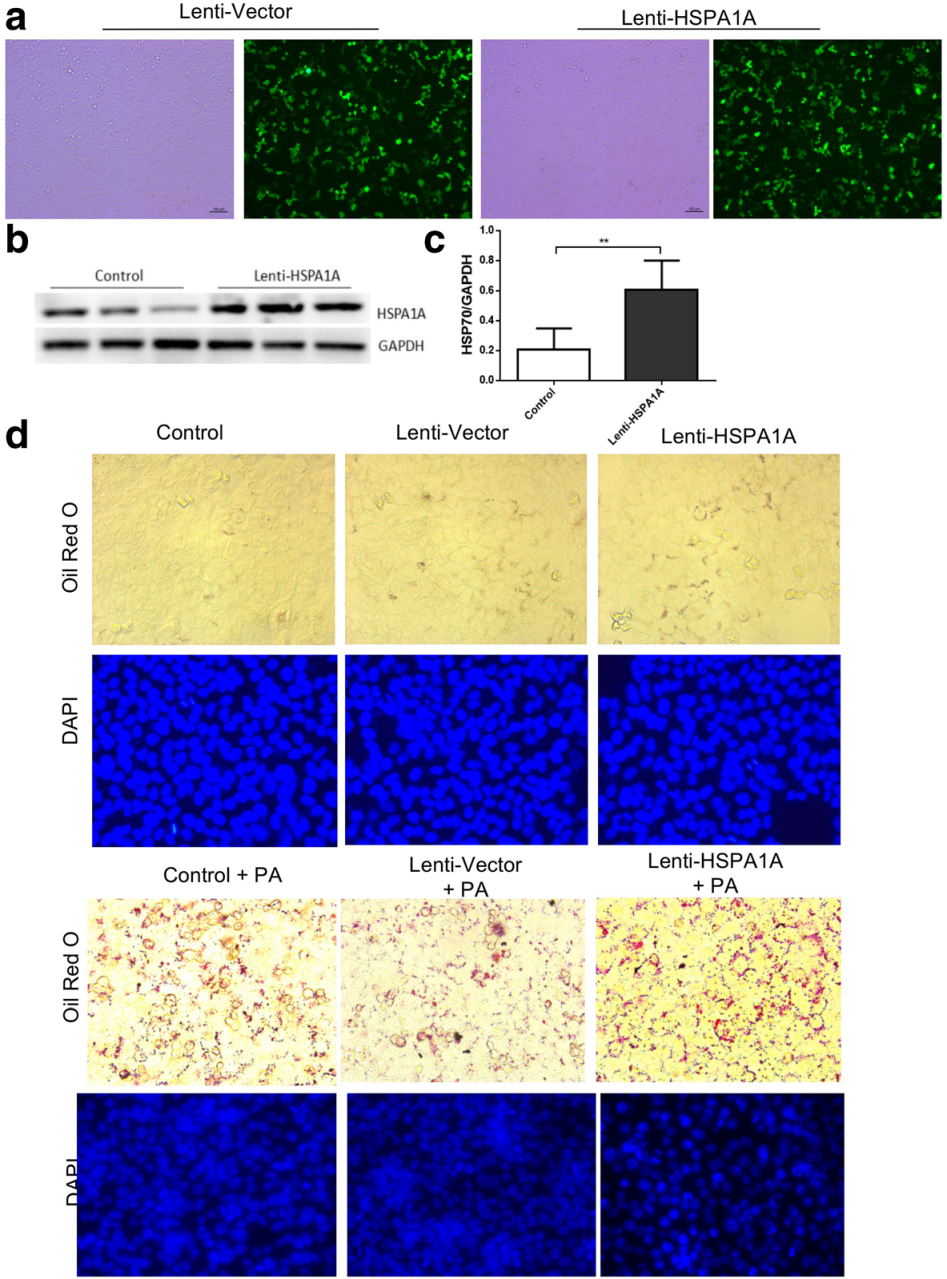
HSP70 overexpression induces fat accumulation and increases the size of lipid droplets. Construction of Lenti-HSPA1A lentiviral vectors for identification of efficient lenti-HSPA1A lentivirus. Schematic representation of lentiviral construction (**a**), HSP70 protein (**b**) expression levels were evaluated by Western blot and normalized to GAPDH levels (**c**). Data are expressed as the mean ± SD. HepG2 cells infected with control or Hsp70-overexpressing lentivirus were treated with or without 0.5 mM PA for 24 h, and lipids were stained with Oil Red O and analysed by microscopy (**d**). Hsp70 overexpression had a small effect on lipid accumulation in HepG2 cells. The cells were infected with an Hsp70-overexpressing lentivirus and then treated with or without PA (0.5 mM) for 24 h. All experiments were repeated three times

To determine whether HSP70 could promote lipid accumulation in HepG2 cells, we established HepG2 cells stably overexpressing human Hsp70. Schematic representation of lentiviral construction (Fig. 2a), HSP70 protein expression levels were evaluated by Western blot and normalized to GAPDH levels (Fig. 2b, c). As shown in Fig. 2d, HSP70 overexpression slightly increased the number of lipid droplets in HepG2 cells compared with cells stably expressing the empty lentiviral vector alone. By contrast, the number of lipid droplets in cells overexpressing HSP70 sharply increased compared with cells expressing the lentiviral vector alone and the control cells upon treatment with PA (Fig. 2d).

### HSPA1A overexpression promotes lipogenesis in HepG2 cells

**Fig. 3:**
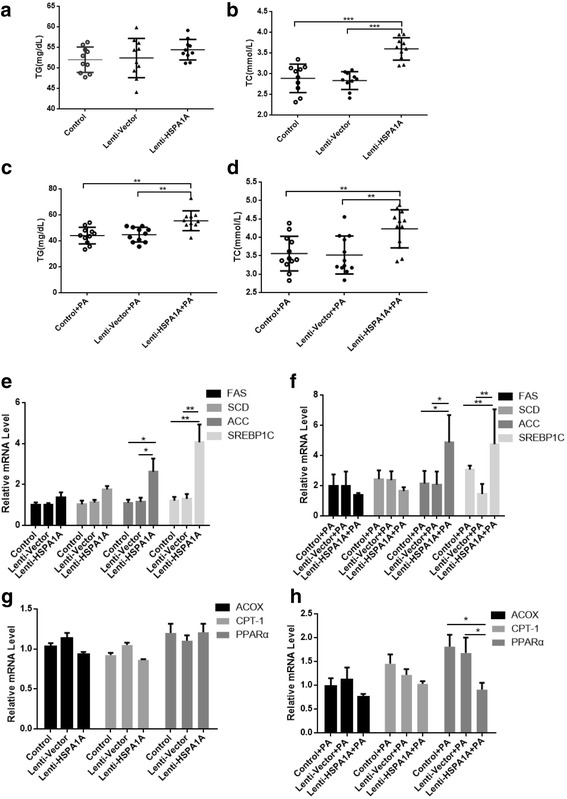
HSPA1A overexpression leads to increased expression of lipogenic enzymes. Cells were analysed for total triglyceride (TG) and total cholesterol (TC) levels (**a**, **b**, **c**, **d**). Data represent the mean ± s.d, ** *P* < 0.01, * *P* < 0.05. Cells were infected with an Hsp70-overexpressing lentivirus and then treated with or without PA (0.5 mM) for 24 h. The mRNA expression levels of FAS, SCD, ACC and SREBP1C as well as the lipid oxidation-related enzymes ACOX, CPT-1, and PPARα were determined by real-time PCR (**e**, **f**, **g**, **h**). All experiments were repeated three times. Data represent the mean ± s.d

Next, the TG and TC levels in the supernatant of HepG2 cells were quantified by ELISA kits. The TG level increased slightly in the supernatant of cells overexpressing HSPA1A (Fig. 3a), while the TC level increased significantly in the supernatant of cells overexpressing HSPA1A (Fig. 3b) compared with that of cells expressing the lentiviral vector alone and the control group. Upon treatment with PA, TG and TC secretion was markedly increased in cells overexpressing HSP70 (Fig. 3c,d).

In addition, the mRNA levels of lipogenic enzymes such as FAS, SCD, ACC and SREBP1C and lipidolysis-related genes such as ACOX, CPT-1, and PPARα were examined by quantitative PCR analysis. ACC and SREBP1C expression increased significantly in HSPA1A-overexpressing cells, regardless of whether they were treated with PA (Fig. 3e, f), and ACOX, CPT-1and PPARα expression was not affected by HSPA1A-overexpressing (Fig. 3g) but PPARα expression decreased in HSPA1A-overexpressing cells treated with PA (Fig. 3h).

### HSPA1A knockdown leads to lipid reduction

**Fig. 4:**
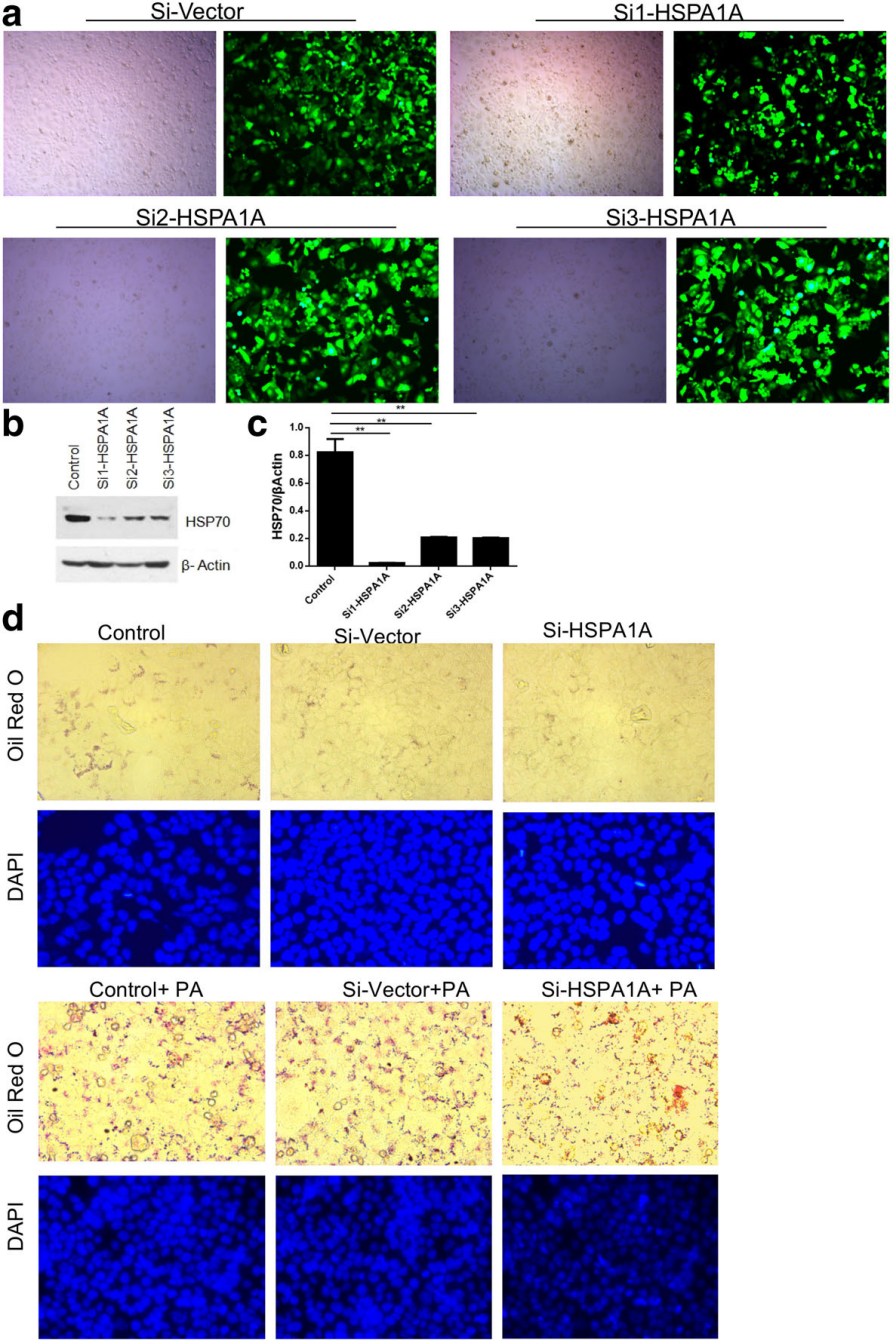
HSPA1A knockdown decreases fat accumulation in NAFLD. HepG2 cells were transfected with vector or HSPA1A siRNA, and the lipids were stained with Oil Red O and analysed by microscopy (**a**, **d**). Hsp70 protein (**b**) expression levels were evaluated by Western blot and normalized to β-actin levels. Data are expressed as the mean ± SD (*n* = 6, each). **P < 0.01 compared with the control group (**b**, **c**)

To further address the role of HSP70 in hepatic lipid accumulation, we knocked down HSP70 in HepG2 cells. As shown in Fig. 4b and c, when HSP70 was knocked down, there was no significant difference in the number of lipid droplets in HSP70-knockdown HepG2 cells compared with cells transfected with a control vector (Fig. 4d). Upon treatment with PA, HSP70-knockdown cells displayed fewer lipid droplets than the control cells (Fig. 4d).

### HSPA1A knockdown leads to decreases in fat synthesizing enzymes

**Fig. 5:**
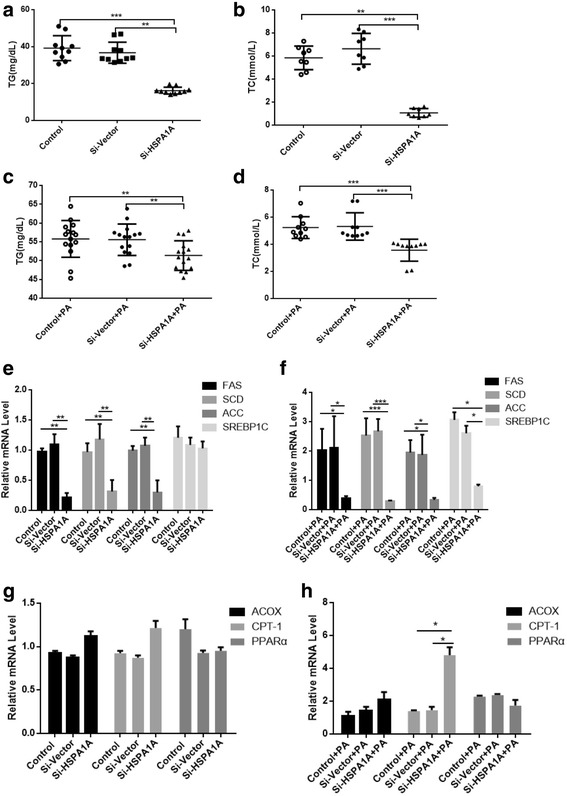
HSPA1A knockdown leads to decreased expression of fat synthesizing enzymes. Cells were analysed for total triglyceride (TG) and total cholesterol (TC) levels (**a**-**b**). The mRNA expression levels of FAS, SCD, ACC, SREBP1C, ACOX, CPT-1, and PPARα were determined by real-time PCR (**e**, **g**). Data represent the mean ± s.d., ** *P* < 0.01, * *P* < 0.05, *** *P* < 0.001. Cells were transfected with either HSPA1A or control siRNA and then treated with PA (0.5 mM) for 24 h. Cellular TG and TC levels were then determined (**c**-**d**). The mRNA expression levels of FAS, SCD, ACC, SREBP1C, ACOX, CPT-1 and PPARα were determined by real-time PCR (**f**, **h**). All experiments were repeated three times. Data represent the mean ± s.d

The TG and TC levels in HepG2 cell supernatants were significantly lower in siHSPA1A-transfected cells compared with control cells, regardless of whether they were treated with PA (Fig. 5a-d).

The mRNA levels of FAS, SCD, ACC, SREBP1C, ACOX, CPT-1 and PPARαin siHSPA1A-transfected HepG2 cells and cells transfected with an empty vector were then examined by quantitative PCR analysis. FAS, SCD and ACC decreased significantly in siHSPA1A-transfected cells as well (Fig. 5e). We also found that upon treatment with PA, the expression of FAS, SCD, ACC and SREBP1C remained low in siHSPA1A-transfected cells (Fig. 5f). Additionally, ACOX, CPT-1and PPARα expression was not affected by HSPA1A-knock down (Fig. 5g) but the expression of CPT-1 increased in siHSPA1A-transfected cells upon treatment with PA (Fig. 5h).

## Discussion

Abnormal accumulation of TG-rich lipid droplets in the liver is a hallmark of NAFLD [7], but the molecular mechanisms driving this phenotype remain unclear. The present study identified a previously unknown function of HSP70 in regulating NAFLD. HSP70 expression is up-regulated in fatty liver, and HSP70 overexpression in HepG2 cells enhanced TG and TC synthesis, in parallel with the upregulation of lipogenic genes, including FAS, SCD and ACC. Thus, we have revealed that HSP70 promotes hepatic steatosis in HepG2 cells.

Lipid accumulation in the liver is associated with ER stress, mitochondrial stress or oxidative stress and impaired autophagy, resulting in lipotoxicity [7–10]. HSPs are a family of proteins that are produced by cells in response to stressful conditions [11, 12]. Hsp90 was found to regulate PPARγ activity in a mouse model of NAFLD, and its overexpression in cells could increase neutral lipid accumulation [5]. Previous studies have revealed that S-nitroso-N-acetylcysteine attenuated liver fibrosis in experimental non-alcoholic steatohepatitis via a mechanism that involved HSP60 downregulation [13]. HSP70, an informative representative of the HSP gene family, was recently found to be decreased in the liver and adipose tissue of obese patients. This decrease may affect HSP70-dependent anti-inflammatory factors and result in increased oxidative stress and insulin resistance in advanced stages of NAFLD [14]. Oxidative stress in the livers of rats fed a HFD was indicated by lower Hsp70 expression, and impaired apoptosis was shown by lower caspase-3 expression [15]. We report here that HSP70 was significantly increased in the livers of obese mice. In vitro, HSP70 expression was markedly induced by PA. Furthermore, HSPA1A overexpression promoted lipid accumulation in HepG2 cells. These results suggested that HSP70 may promote lipid accumulation in the early stages of NAFLD.

The majority of hepatic lipids in NAFLD are stored in the form of TGs, which are synthesized from free fatty acids (FFAs). TG accumulation thus depends on the presence of FFAs in the liver and their disposal. Lipogenic genes such as FAS, SCD, and ACC are directly up-regulated during lipid accumulation [16–18]. FAS is a multi-enzyme, and its function is to catalyse the de novo synthesis of fatty acids [19, 20]. Silva Veiga FM found that the expression of PPARγ as well as its target genes SREBP-1, GK and FAS increased in a HFD group compared with a control group [21]. SCD-1 is also a key enzyme for the synthesis of monounsaturated fatty acids [22]. Moreover, ACC is the most well-known substrate for AMPK and catalyses the carboxylation of acetyl-CoA to malonyl-CoA, which is the limiting step in fatty acid biosynthesis [23]. SREBP1c is a key transcription factor of lipid metabolism and energy storage [24] by inducing lipogenic enzymes, including ACC, FAS and SCD [25]. PPARα upregulates several genes involved in oxidative lipid metabolism, including CPT1, PDK4, CYP4A and ACOX1 [26, 27]. It functions as a major regulator of fatty acid β-oxidation in liver, Our results suggest that HSP70 overexpression in HepG2 cells enhanced lipid synthesis, in parallel with the upregulation of lipogenic genes, including SREBP1C,FAS, SCD and ACC. It may be speculated that HSP70 increases SREBP1C, which upregulates FAS, SCD, and ACC and promotes lipogenesis. By contrast, HSP70 knockdown decreased lipid accumulation and the expression of FAS, SCD and ACC, but had little effect on PPARα in HepG2 cells. So it is suggested that HSP70 promote lipid accumulation in hepatocytes via lipogenesis and not decreased oxidation of fatty acid. However, our study does not exclude other additional mechanisms mediating the function of HSP70 in lipid accumulation in hepatocytes.

## Conclusion

In conclusion, this study establishes the role of HSP70 in NAFLD. Furthermore, unpublished data in our laboratory have also revealed that mice with Hsp70 knockdown exhibit decreased insulin sensitivity. Thus, it is tempting to speculate that HSP70 knockdown may also protect against NAFLD. Our findings suggest that HSP70 promotes lipogenesis in HepG2 cells, making it an attractive drug target, as pharmacological manipulation of Hsp70 could have potential uses in the treatment of metabolic disorders such as diabetes and liver steatosis, among others.

## Abbreviations

ACC: Acetyl-CoA carboxylase
ELISA: Enzyme-linked immunosorbent assay
FAS: Fatty acid synthase
HSP70: Heat shock protein 70
HSPs: Heat shock proteins
NAFLD: Non-alcoholic fatty liver disease
PA: Palmitic acid
PBS: Phosphate-buffered saline
SCD: Stearoyl-CoA desaturase
TC: Total cholesterol
TG: Triglyceride
